# P042 Beyond headache and visual loss: systemic and neuropsychiatric presentations of giant cell arteritis

**DOI:** 10.1093/rap/rkag113.072

**Published:** 2026-11-03

**Authors:** Rehab Mohammed, Usama Al Siyabi, Kirsty Levasseur, Anu Desai

**Affiliations:** Rheumatology Department, The Dudley Group, Dudley, United Kingdom; Rheumatology Department, The Dudley Group, Dudley, United Kingdom; Rheumatology Department, The Dudley Group, Dudley, United Kingdom; Rheumatology Department, The Dudley Group, Dudley, United Kingdom

## Abstract

**Introduction:**

Giant cell arteritis (GCA) is a granulomatous large-vessel vasculitis affecting the aorta and its major branches, typically occurring in adults over 50 years. It classically presents with cranial symptoms including headache, scalp tenderness, jaw claudication and visual disturbance, but may also involve extra-cranial vessels with systemic manifestations. Increasing use of vascular ultrasound and cross-sectional imaging has broadened recognition of atypical phenotypes that may mimic infection, malignancy, inflammatory arthritis or neurological disease. We report three cases of imaging-confirmed GCA with unusual systemic and neurological presentations, including polyserositis, inflammatory polyarthritis and neuropsychiatric features with vertebral artery involvement.

**Case description:**

Case 1: A 76-year-old man presented with constitutional symptoms, cough and markedly raised inflammatory markers. CT thorax, abdomen and pelvis demonstrated bilateral pleural effusions, mild ascites and pericardial inflammation consistent with polyserositis. Extensive infection and malignancy work-up was negative. Subsequent vascular assessment confirmed GCA. Prednisolone with gastric and bone protection resulted in rapid clinical improvement.

Case 2: A 90-year-old man presented with a two-month history of inflammatory polyarthritis affecting metacarpophalangeal joints, wrists, shoulders and lower limbs, with prolonged morning stiffness, fatigue, weight loss and night sweats. Examination showed active synovitis with ten swollen and tender joints. Inflammatory markers were modestly elevated (ESR 33 mm/hr, CRP 8 mg/L). Temporal headaches subsequently developed. Urgent temporal artery ultrasound demonstrated bilateral halo signs confirming GCA. Imaging excluded malignancy. High-dose corticosteroids led to marked improvement in systemic and joint symptoms.

Case 3: A 75-year-old man presented with headache, neck pain and progressive visual loss culminating in left anterior ischaemic optic neuropathy. Temporal artery ultrasound confirmed GCA with vertebral artery involvement. MRI demonstrated V3/V4 vertebral artery abnormalities suggestive of partial thrombosis. Following intravenous methylprednisolone, he developed acute visual hallucinations in the context of active vasculitis and systemic illness. Symptoms were transient and improved with medical stabilisation and corticosteroid tapering. No further visual deterioration occurred during follow-up.

**Discussion:**

These cases illustrate that extra-cranial and neurological manifestations may dominate the clinical presentation of GCA, delaying recognition of the underlying vasculitis.

Polyserositis is an uncommon manifestation of GCA. In Case 1, pleural, pericardial and peritoneal involvement prompted extensive exclusion of infection and malignancy before a unifying diagnosis was established. Recognition of serositis as part of the disease spectrum may reduce diagnostic delay.

Case 2 demonstrates inflammatory polyarthritis as a presenting feature of GCA, which may closely mimic elderly-onset rheumatoid arthritis or polymyalgia rheumatica. Modest inflammatory marker elevation further contributed to diagnostic uncertainty, with temporal artery ultrasound proving decisive.

Case 3 highlights vertebral artery involvement associated with acute visual hallucinations. These symptoms are rarely reported in GCA and are likely multifactorial in origin in the context of active vasculitis and systemic illness. This distinction is important to avoid misattribution to primary neurological or psychiatric disease.

Across all cases, vascular imaging was central to diagnosis following exclusion of alternative causes. These cases emphasise that GCA may present without classical cranial symptoms and with predominant systemic or neurological features. Awareness of such atypical phenotypes is essential to facilitate early diagnosis and prevent irreversible complications.

**Key learning points:**

1. GCA may present with extra-cranial systemic and neurological manifestations that overshadow classical cranial features.

2. Polyserositis and peripheral inflammatory arthritis are uncommon but recognised manifestations of GCA.

3. Neuropsychiatric symptoms, including visual hallucinations, may occur in patients with GCA in the context of active disease and systemic illness.

4. Temporal artery ultrasound and vascular imaging are central to diagnosis in atypical presentations.

5. Awareness of atypical phenotypes may reduce diagnostic delay and prevent irreversible complications.

